# Defining the RNaseH2 enzyme-initiated ribonucleotide excision repair pathway in Archaea

**DOI:** 10.1074/jbc.M117.783472

**Published:** 2017-04-03

**Authors:** Margaret R. Heider, Brett W. Burkhart, Thomas J. Santangelo, Andrew F. Gardner

**Affiliations:** From ‡ New England Biolabs, Inc., Ipswich, Massachusetts 01938 and; the § Department of Biochemistry and Molecular Biology, Colorado State University, Fort Collins, Colorado 80521

**Keywords:** archaea, DNA damage, DNA repair, DNA replication, DNA synthesis, enzyme kinetics, pre-steady-state kinetics, RNaseH2, ribonucleotide excision repair

## Abstract

Incorporation of ribonucleotides during DNA replication has severe consequences for genome stability. Although eukaryotes possess a number of redundancies for initiating and completing repair of misincorporated ribonucleotides, archaea such as *Thermococcus* rely only upon RNaseH2 to initiate the pathway. Because *Thermococcus* DNA polymerases incorporate as many as 1,000 ribonucleotides per genome, RNaseH2 must be efficient at recognizing and nicking at embedded ribonucleotides to ensure genome integrity. Here, we show that ribonucleotides are incorporated by the hyperthermophilic archaeon *Thermococcus kodakarensis* both *in vitro* and *in vivo* and a robust ribonucleotide excision repair pathway is critical to keeping incorporation levels low in wild-type cells. Using pre-steady-state and steady-state kinetics experiments, we also show that archaeal RNaseH2 rapidly cleaves at embedded ribonucleotides (200-450 s^−1^), but exhibits an ∼1,000-fold slower turnover rate (0.06–0.17 s^−1^), suggesting a potential role for RNaseH2 in protecting or marking nicked sites for further processing. We found that following RNaseH2 cleavage, the combined activities of polymerase B (PolB), flap endonuclease (Fen1), and DNA ligase are required to complete ribonucleotide processing. PolB formed a ribonucleotide-containing flap by strand displacement synthesis that was cleaved by Fen1, and DNA ligase sealed the nick for complete repair. Our study reveals conservation of the overall mechanism of ribonucleotide excision repair across domains of life. The lack of redundancies in ribonucleotide repair in archaea perhaps suggests a more ancestral form of ribonucleotide excision repair compared with the eukaryotic pathway.

## Introduction

The fidelity and efficiency of genome replication is essential in all domains of life. DNA polymerases and the associated multiprotein replication machinery must therefore be remarkably accurate in copying the template DNA strand. In addition to selecting complementary base pairs, all DNA polymerases must also discriminate between deoxyribonucleoside triphosphates (dNTPs) and ribonucleoside triphosphates (rNTPs) during DNA replication. DNA polymerases limit rNTP binding in the active site by a steric clash between the rNTP 2′-hydroxyl and an amino acid side chain (1–3). However, all DNA polymerases face a constant challenge to exclude rNTPs *in vivo* as the intracellular concentration of rNTPs exceeds that of dNTPs by 30–1000-fold (4–9). Therefore, ribonucleotide monophosphates (rNMPs)^2^ are likely the most commonly incorporated noncanonical nucleotides in the cell (6, 9–12). For example, eukaryotic replicative DNA polymerases incorporate rNTPs both *in vitro* and *in vivo* at ∼1 rNMP per 1,000 bp and *Escherichia coli* DNA PolIII at 1 rNMP per 2,300 bp (6, 9, 10, 12).

Once incorporated by DNA polymerases, embedded rNMPs have severe consequences for genome integrity. The reactive 2′-hydroxyl group on rNMPs renders the strand highly susceptible to cleavage, making RNA much less stable than DNA for the storage of genetic information (13, 14). Furthermore, inclusion of rNMPs alters the structural conformation of DNA from B-form to A-form, creating problems for DNA polymerases, which require a specific B-form helical geometry for accurate and efficient replication (15–17). Consistent with these observations, mammalian cells that accumulate rNMPs exhibit strand breakage, chromosomal rearrangements, replication fork stalling, and activation of DNA damage response pathways (18). Additionally, mouse strains defective in ribonucleotide repair are embryonic lethal and humans who accumulate embedded rNMPs suffer from Aicardi-Goutieres syndrome, a severe autoimmune disease (18, 19).

To protect genome stability, cells require repair mechanisms for removing these unstable, embedded rNMPs. A specific repair pathway termed ribonucleotide excision repair (RER) serves this function and was recently described in eukaryotic and bacterial systems (11, 20). In both cases, RER begins with specific nicking at a single embedded rNMP by RNaseH2. A DNA polymerase extends the nick, displacing the rNMP to leave a flap that is cleaved by a nuclease and then sealed by DNA ligase. Although they follow a similar pathway, eukaryotes and bacteria make use of different enzymatic machineries to carry out each step of the repair (11, 20). The essential nature of ribonucleotide removal is highlighted by the fact that eukaryotes also possess a redundant, minor RER pathway initiated by topoisomerase I, although this pathway likely induces mutagenic effects of its own (21, 22).

An RER pathway has not been described in archaea. It is not yet known whether archaea require an RER pathway and if they do, the mechanism and enzymes involved have not yet been defined. Many components of the archaeal replication and repair machinery have been identified and although many of these proteins show strong homology to eukaryotes, there are also archaea-specific proteins that may constitute unique pathways and mechanisms. For example, all archaea express a Family B polymerase (PolB) but many also express an essential, archaea-specific Family D polymerase (PolD) and the division of labor between these two polymerases in DNA replication and repair is not yet clear. Our study makes use of two closely related, hyperthermophilic archaea: *Thermococcus kodakarensis* (Tko) for genetics and *Thermococcus* sp. 9°N (9°N) for biochemistry (reviewed in Ref. 23). Using these organisms as models, we reconstituted the archaeal RER pathway using a combination of genetics, biochemistry, and enzyme kinetics. We identified RNaseH2, PolB, Fen1, and DNA ligase as the essential enzymes for this process. These studies not only define RER for the first time in archaea, but reveal conservation of the RER mechanism across domains of life and shed light on the specific roles for the archaeal DNA polymerases, PolD and PolB, in *Thermococcus*.

## Results

### rNMPs are incorporated during DNA replication in Thermococcus

**Figure 1.**
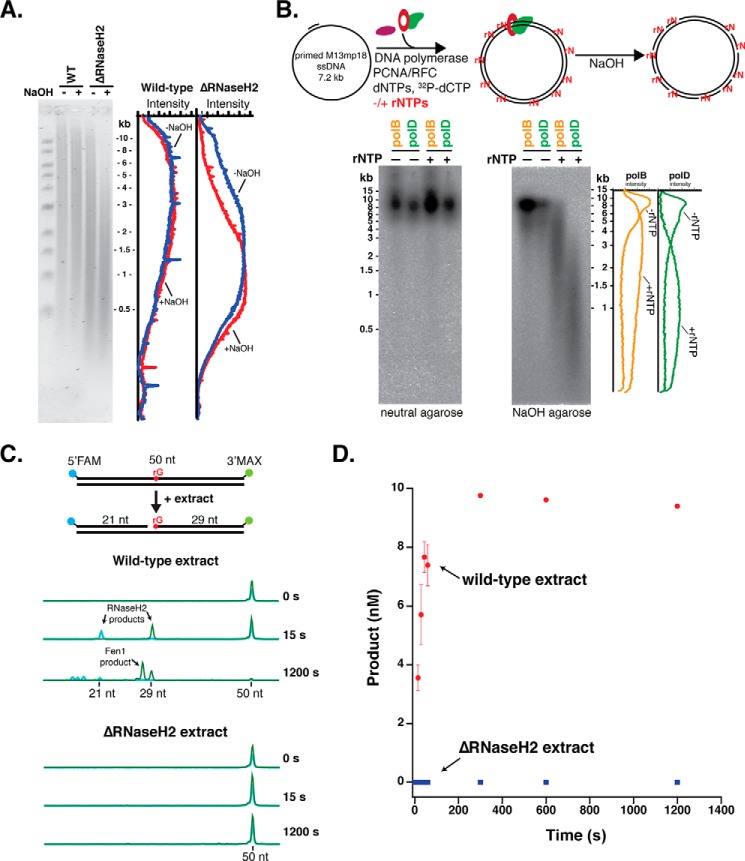
**Archaea require an RNaseH2-initiated RER pathway.** *A*, genomic DNA from wild-type and ΔRNaseH2 *T. kodakarensis* (*Tko*) cells was treated with either 0.3 m NaCl or 0.3 m NaOH, separated on a 1% alkaline-agarose gel, and visualized using SYBR Gold staining. The fluorescence intensity distribution was quantified using ImageQuant software. *B,* primed M13mp18 ssDNA was fully extended by either *Thermococcus* sp. 9°N PolB or PolD with dNTPs or dNTPs/excess rNTPs (see “Experimental procedures”) with [^32^P]dCTP in place of dCTP for phosphorimaging. Full extension products were visualized by neutral agarose and rNMP incorporation was assessed by 0.3 m NaOH treatment and alkaline-agarose electrophoresis. *C,* RNaseH2 activity in Tko extracts was monitored by CE using a 50-bp dsDNA substrate with an embedded rGMP nucleotide, a 5′-FAM label, and a 3′-MAX label. Reactions were carried out over a time course from 15 s to 20 min at 60 °C. A subset of representative CE traces are shown indicating the formation of 21- and 29-nt RNaseH2 products. 3′-MAX-labeled Fen1 flap cleavage products (<29 nt) are also observed. *D,* the formation of the 29-nt MAX product was quantified over time for both wild-type (*red*) and ΔRNaseH2 (*blue*) extracts. Data are the average of three biological replicates and *error bars* indicate standard deviation.

We examined the *in vivo* frequency of rNMP incorporation by DNA polymerases during archaeal chromosome replication using a Tko strain deleted for RNaseH2. In both eukarya and bacteria, RER is initiated by the enzyme RNaseH2, which nicks DNA 5′ to a single, embedded rNMP (11, 20, 24–26). Archaea express a monomeric RNaseH2 enzyme homologous to the catalytic subunit of the eukaryotic enzyme (27, 28). We therefore predicted that it may have the same function in archaea. The presence of rNMPs in DNA can be visualized using alkaline-agarose electrophoresis. The reactive 2′-hydroxyl group on ribonucleotides render DNA highly sensitive to strand cleavage with NaOH, resulting in fragmented DNA that migrates faster on a denaturing alkaline-agarose gel. In wild-type Tko, most rNTPs incorporated by a DNA polymerase are presumably repaired. Indeed, wild-type Tko genomic DNA is minimally sensitive to alkaline hydrolysis suggesting that few genomic rNMPs remain after repair (Fig. 1*A*). In the absence of RNaseH2 (ΔRNaseH2), rNMPs incorporated by DNA polymerases remain in genomic DNA and reveal the underlying frequency of rNMP incorporation by DNA polymerases. Tko ΔRNaseH2 genomic DNA migrated as lower molecular weight fragments indicative of alkali-sensitive ribonucleotide sites that remain in the genomic DNA (Fig. 1*A*). The size distribution of the fragments indicates an approximate ribonucleotide incorporation frequency of 1 in 1,500 nucleotides in the absence of RNaseH2, which corresponds to at least 1,000 genomic rNMPs incorporated per genome copy. These results demonstrate that archaeal DNA polymerases incorporate ribonucleotides in genomic DNA and that RNaseH2 is an important enzyme for repair.

The frequency of rNTP incorporation was also probed by *in vitro* synthesis with *Thermococcus* sp. 9°N DNA polymerases B and D. We monitored the extension of primed M13 DNA with [α-^32^P]dCTP in the presence of dNTPs or predicted concentrations of dNTPs and rNTPs. Similar to the previous experiment, sensitivity to NaOH hydrolysis was a measure of rNTP incorporation (Fig. 1*B*). Both polymerases fully extended M13 DNA under both conditions as assayed by neutral agarose electrophoresis (Fig. 1*B*, *left*). Alkaline hydrolysis showed that both polymerases incorporate rNTPs at a high frequency with 1 in 2,500 and 1 in 1,500 nucleotides for PolB and PolD, respectively (Fig. 1*B*, *right*) (6). Together these results confirm that rNTPs are incorporated during synthesis by PolB and PolD both *in vivo* and *in vitro*.

### RNaseH2-initiated repair is the major pathway in Thermococcus for repairing embedded rNMPs

Eukaryotes use RNaseH2-initiated RER as the primary pathway for excising embedded rNMPs, however, due to the critical need for this repair, they also possess a redundant pathway that instead begins with incision by topoisomerase I at the ribonucleotide (9). As in yeast, RNaseH2 is not an essential gene in archaea, and deletion of this gene (*tk_rs03985*) showed no detectable growth defects in *Thermococcus*.^3^ Therefore, we next investigated whether there might be a redundant enzyme in *Thermococcus* that nicks DNA at rNMPs other than RNaseH2. Although there were abundant rNMPs in the genomic DNA of cells deleted for RNaseH2, loss of a compensatory enzyme that nicks at rNMPs could result in an even greater load of embedded, toxic rNMPs. To test whether a different enzyme is capable of the same cleavage reaction as RNaseH2, we designed a substrate to mimic the product resulting from polymerase incorporation errors: a DNA strand containing a single embedded rNMP. Using a dual-labeled (5′-FAM and 3′-MAX) 50-bp double-stranded DNA substrate with a single, embedded rGMP nucleotide, we monitored *Thermococcus* extracts for cleavage activity at the rGMP over time using capillary electrophoresis (CE) (Fig. 1*C*) (29). We quantified the appearance of the 3′-MAX product at each time point (Fig. 1*D*), and observed rapid conversion of the 50-bp, dual-labeled DNA substrate to individual DNA fragments with wild-type extracts (Fig. 1, *C* and *D*). However, ΔRNaseH2 extracts incubated even for extended time points showed no cleavage activity at rG (Fig. 1, *C* and *D*). Addition of purified *Thermococcus* sp. 9°N RNaseH2 in the presence of ΔRNaseH2 cell extracts yields the expected cleavage products, indicating no inhibitory activities present in the extract (data not shown). These results, combined with the lack of repair of genomic DNA in ΔRNaseH2 strains, indicate that RNaseH2 is the major enzyme to initiate repair of embedded rNMPs and that there is no detectable enzyme activity in *Thermococcus* that can compensate for it.

### Kinetic analysis of Thermococcus RNaseH2

**Figure 2.**
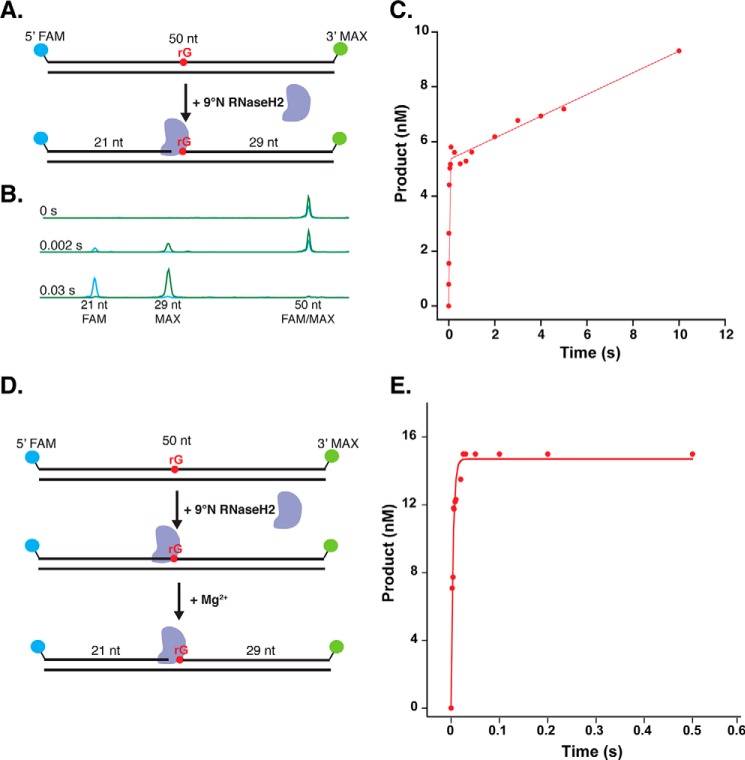
**RNaseH2 cleavage is not the rate-limiting step in RER.** *A*, for steady-state kinetics, a 5-fold excess of 50 bp, 5′-FAM, 3′-MAX-labeled rGMP RER substrate was rapidly mixed with purified *Thermococcus* sp. 9°N RNaseH2 in an RQF instrument at 60 °C. The reaction was quenched with 50 mm EDTA. *B,* the conversion of the 50-nt, dual-labeled rG substrate to 5′-FAM and 3′-MAX cleavage products was monitored over time using CE. A subset of representative traces from a time course are shown. *C,* the yield of 5′-FAM product was graphed as a function of time and fit to Equation 1 to obtain *k*_ss_ = 0.06 s^−1^ and the active enzyme concentration (*A*) = 5.5 nm. A representative plot for the rG substrate is shown and replicates for rG and the other ribonucleotide substrates are shown in supplemental Fig. S1. *D,* pre-steady state kinetics were performed with purified *Thermococcus* sp. 9°N RNaseH2 chelated for metal ion, pre-bound to the 50-bp RER substrates with different embedded rNMPs. The enzyme was in 3-fold excess to the DNA substrate. The pre-bound DNA-RNaseH2 complex was rapidly mixed with buffer containing MgCl_2_ in an RQF at 60 °C and quenched with 50 mm EDTA. Cleavage products were visualized by CE. *E,* the yield of 5′-FAM product was graphed as a function of time and fit to Equation 2 to obtain *k*_cleavage_ for rG, rA, rC, and rU (222, 196, 447, and 426 s^−1^). A representative plot for the rG substrate is shown. Technical replicates of the pre-steady-state experiments for the rG and other ribonucleotide substrates are shown in supplemental Fig. S2.

Because ∼1,000 rNTPs are incorporated each time the 2-Mb genome is replicated, the activity of *Thermococcus* RNaseH2 on embedded rNMPs must be efficient. Tko extract experiments showed rapid cleavage activity by RNaseH2 on embedded ribonucleotides (Fig. 1*C*). Using steady-state and pre-steady-state kinetic experiments, we aimed to quantify the rate of cleavage of RNaseH2 on an embedded rNMP, identify the substrate preferences of RNaseH2 among the different potential embedded rNMPs, and determine what might be the rate-limiting steps in RER. First, we performed steady-state kinetic experiments to determine the turnover rate of *Thermococcus* sp. 9°N RNaseH2. To monitor enzyme turnover, we used the fluorescently-labeled 50-bp, double-stranded DNA substrate containing a single embedded rGMP in the labeled strand (Fig. 2*A*, Table 1) in 5-fold excess to the enzyme and monitored the appearance of the 5′-FAM 21-nt and 3′-MAX 29-nt cleavage products over time using a rapid quench flow instrument (RQF) and CE (Fig. 2*B*) (29). The concentration of the 5′-FAM product was plotted as a function of time and the steady-state rate (*k*_ss_) and active enzyme concentration (A) were determined. The active enzyme concentration, 5.5 nm (Fig. 2*C*), was used to calculate the concentration of enzyme used in subsequent pre-steady-state kinetic experiments. The steady-state turnover rates (*k*_ss_) were 0.06, 0.08, 0.17, and 0.14 s^−1^ for rG, rA, rC, and rU substrates, respectively (Fig. 2*C*, supplemental Fig. S1, and Table 2).

**Table 1:** **Oligonucleotide substrates used in this study**

Name	Sequence
50-nt 5′-FAM/3′-MAX rGMP	5′-FAM-GGG AGC GTC GAC AGC TTG GAT **rG**AG TGC CAC TTG TCT ACG GCT ATG CCT TA-MAX-3′
50-nt rGMP complement	5′-TAA GGC ATA GCC GTA GAC AAG TGG CAC TCA TCC AAG CTG TCG ACG CTC CC-3′
50-nt 5′-FAM rCMP	5′-FAM-GGG AGC GTC GAC AGC TTG GAT **rC**AG TGC CAC TTG TCT ACG GCT ATG CCT TA-3′
50-nt rCMP complement	5′-TAA GGC ATA GCC GTA GAC AAG TGG CAC TGA TCC AAG CTG TCG ACG CTC CC-3′
50-nt 5′-FAM rAMP	5′-FAM-GGG AGC GTC GAC AGC TTG GAT **rA**AG TGC CAC TTG TCT ACG GCT ATG CCT TA-3′
50-nt rAMP complement	5′-TAA GGC ATA GCC GTA GAC AAG TGG CAC TTA TCC AAG CTG TCG ACG CTC CC-3′
50-nt 5′-FAM rUMP	5′-FAM-GGG AGC GTC GAC AGC TTG GAT **rU**AG TGC CAC TTG TCT ACG GCT ATG CCT TA-3′
50-nt rUMP complement	5′-TAA GGC ATA GCC GTA GAC AAG TGG CAC TAA TCC AAG CTG TCG ACG CTC CC-3′
44-nt 5′-MAX RER	5′-MAX-CGC CAG GGT TTT CCC AGT CAC GAC GTT GTA AAA CGA CGG CCA GT-3′
90-nt 3′-FAM RER with 5′phosphorylated rGMP	5′-Phosph-**rG**CC AAG CTT GCA TGC CTG CAG GTC GAC TCT AGA GGA TCC CCG GGT ACC GAG CTC GAA TTC GTA ATC ATG GTC ATA GCT GTT TCC TGT GTG-FAM-3′

**Table 2:** **9°N RNaseH2 kinetic parameters**

Substrate	*k*_ss_	*k*_cleavage_
rG	0.06 ± 0.02 s^−1^	220 ± 22 s^−1^
rA	0.08 ± 0.02 s^−1^	200 ± 12 s^−1^
rU	0.17 ± 0.02 s^−1^	430 ± 44 s^−1^
rC	0.14 ± 0.02 s^−1^	450 ± 37 s^−1^

Pre-steady-state kinetic experiments were performed to determine the rate of cleavage for RNaseH2 and whether this rate varied for each of the four possible embedded ribonucleotides. In all pre-steady-state experiments, the enzyme was in 3-fold excess to the substrate. To accurately quantify the rate of chemistry, RNaseH2 was pre-bound to the DNA substrate and the reaction was triggered by rapid mixing with buffer containing MgCl_2_ using RQF (Fig. 2*D*). The concentration of the 5′-FAM product detected using CE was graphed as a function of time and fit to the pre-steady-state equation to obtain the rate of cleavage (Fig. 2*E*). RNaseH2 showed a rapid rate of cleavage with 220, 200, 450, and 430 s^−1^ for rG, rA, rC, and rU substrates, respectively (supplemental Fig. S2 and Table 2), revealing a slight preference for pyrimidines compared with purines. This contrasted with the human RNaseH2 enzyme, which cleaves all rN substrates but showed a slight preference for purines (25). Given that the rate of chemistry is more than 1,000 times faster than the turnover rate, this suggests that a step after chemistry, likely product release, is rate-limiting.

### Defining the enzymes required for RER in vivo

**Figure 3.**
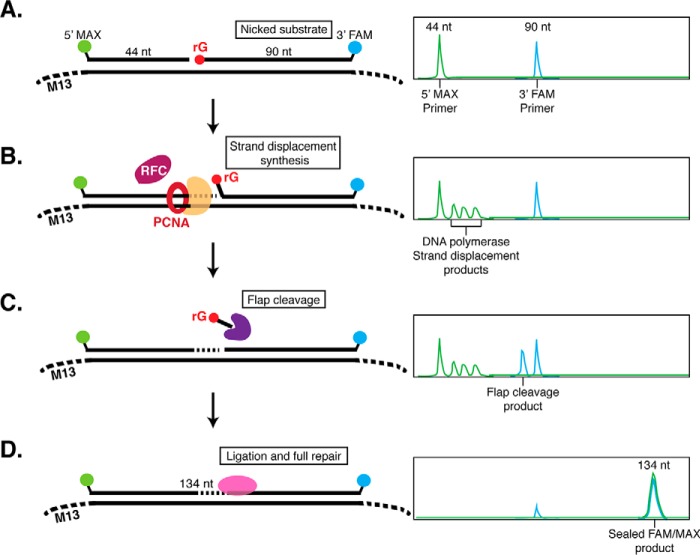
**Dual-label fluorescence assay to monitor post-RNaseH2 RER by capillary electrophoresis.** *A,* the RER substrate was generated by annealing a 44-nt (5′-MAX labeled) oligonucleotide and a 90-nt (3′-FAM labeled with a 5′-phosphate-rGMP) oligonucleotide to ssM13 DNA. Annealed oligos form a DNA substrate containing a nick with 3′-OH and 5′-phosphate-rG termini to mimic DNA nicked by RNaseH2. On CE, the double-stranded DNA is denatured and ssDNA oligonucleotides can be visualized individually. On the *right* is a hypothetical CE trace representing the expected result with two individual MAX and FAM oligonucleotide peaks. *B,* when a strand-displacing DNA polymerase is added, 5′-MAX-labeled strand displacement products larger than 44-nt can be observed. *C,* when a flap endonuclease is added, 3′-FAM products smaller than 90-nt (predominantly by 1–2 nt) are observed. *D,* full sealing and repair by ligation results in a FAM- and MAX-labeled 134-nt product.

After RNaseH2 nicks 5′ to an rNMP, additional enzymes are required to complete repair. To identify and characterize the other enzymes required for RER in *Thermococcus*, we designed a CE repair assay using a dual-labeled DNA substrate that mimics the product of RNaseH2 cleavage (Fig. 3*A*). Two fluorescently-labeled oligonucleotides are annealed to single-stranded M13 DNA: a 44-nt primer and a 90-nt primer with a 5′ phosphate-rGMP. Before addition of extract or purified components, the substrate oligonucleotides appear as two distinct fluorescent peaks of 44- and 90-nt on CE (Fig. 3*A*). Over time, repair intermediates such as extension of the 44-nt primer by DNA polymerase strand displacement (Fig. 3*B*) or flap cleavage of the 90-nt primer (Fig. 3*C*), can be observed. Finally, a completely repaired product is a dual-labeled, sealed DNA product (134 nt) (Fig. 3*D*). As a control to confirm repair, adding purified RNaseH2 at the end of the reaction did not result in detectable rNMP cleavage products, indicating that the rNMP was repaired and replaced with a dNMP and not simply sealed by Ligase (supplemental Fig. S3).

**Figure 4.**
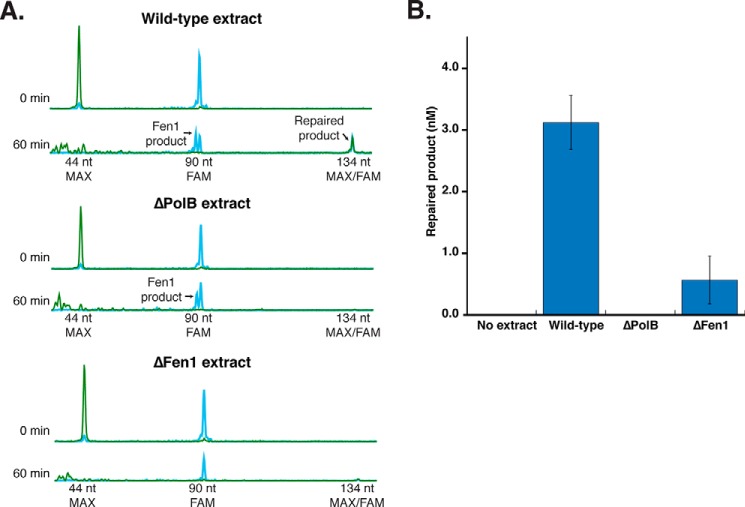
***Thermococcus* extracts reveal critical roles for PolB and Fen1 in RER.** *A,* the substrate depicted in Fig. 3 was incubated with cell extracts prepared from Tko wild-type, Tko ΔPolB, and Tko ΔFen1 strains over a time course from 0 to 60 min at 60 °C (subset of representative traces shown). Over time the overall signal of the substrate peaks (44-nt MAX and 90-nt FAM) decrease due to substrate conversion to repaired product (134-nt FAM/MAX) as well as competing enzymatic activities. *B,* the amount of repaired product at 60 min was quantified for each extract. The data shown are the average of four independent experiments with S.D.

First, we incubated this RER substrate at 10 nm with *Thermococcus* cell extracts from wild-type and mutant strains to identify putative pathway components. All extracts used were normalized by protein concentration and Ligase activity, ensuring that the extracts were otherwise functional. Wild-type extracts repaired the RER substrate within 60 min, although the overall level of repaired product was low (∼3 nm) due to competing nuclease activities in extracts (Fig. 4, *A* and *B*). Extracts deleted for Fen1 (*tk_rs03985*) and PolB (*tk_rs00010*) showed greatly reduced (ΔFen1) or no (ΔPolB) repair (Fig. 4, *A* and *B*). Addition of purified *Thermococcus* sp. 9°N Fen1 and PolB to extracts deleted for these genes complemented RER indicating that these were the key activities lacking and preventing the complete repair of this substrate (supplemental Fig. S4). These results indicate that Fen1 and PolB play critical roles in RER.

### Reconstitution of archaeal RER in vitro

**Figure 5.**
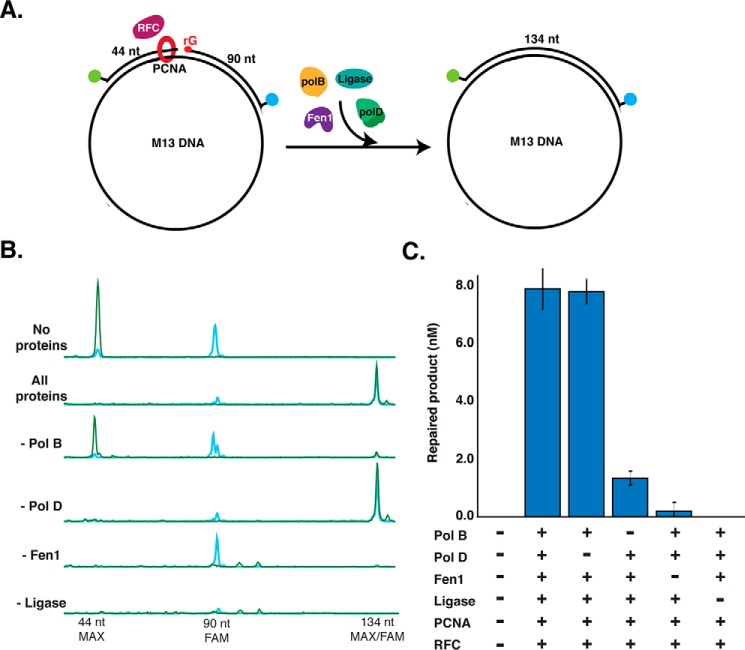
**Archaeal RER reconstituted *in vitro*.** *A,* reaction schematic. The substrate depicted in Fig. 3 is incubated with purified *Thermococcus* sp. 9°N proteins including PCNA, RFC, and different combinations of PolB, PolD, Fen1, and DNA ligase. Reactions were incubated over a time course from 0 to 30 min at 60 °C and repair was monitored by appearance of the 134-nt FAM/MAX-labeled DNA product by CE. *B,* representative CE traces for each reaction condition at the 30-min time point are shown. *C,* quantification of the conversion of 90-nt FAM substrate to 134-nt FAM/MAX product at the 30-min time point. Data are the average of three independent experiments with S.D.

Experiments with Tko extracts suggested key roles for PolB and Fen1 in the RER pathway. Next we aimed to reconstitute the RER pathway *in vitro* using the same DNA substrate as before (Fig. 3) but with purified *Thermococcus* sp. 9°N proteins to identify the minimal required repair machinery. Using all of the components (PolB, PolD, Fen1, Ligase, PCNA, and RFC), repaired product was detectable within 5 min (data not shown) and complete repair occurred within 30 min (Fig. 5). The same level of repair was observed when PolD was absent from the reaction mixture, but omitting PolB, Fen1, or Ligase either greatly reduced (PolB) or eliminated (Fen1 and Ligase) repair of the DNA substrate (Fig. 5, *B* and *C*). Similar to previous studies on the effect of PCNA on Tko replication, PCNA and RFC were not required for repair under these conditions and no requirement was observed until salt concentrations were raised to greater than 100 mm NaCl (30) (data not shown). Repair reactions supported by PolD in the absence of PolB may be due to the low level of strand displacement activity by this polymerase or due to DNA breathing at this temperature driving flap formation and cleavage by Fen1 followed by synthesis and ligation. In the absence of Ligase, both MAX and FAM signals decreased (Fig. 5*B*). PolB extends the 5′-MAX-labeled primer (*green*) and displaces the downstream 3′-FAM-labeled strand (*blue*) creating a 5′ flap that is sequentially cleaved by Fen1. PolB continues to extend the 5′-MAX-primer using M13 as a template strand and synthesizes long products. When the synthesis length is longer than the CE detection range (up to 800 nt), the MAX signal disappears from view. In summary, following DNA nicking by RNaseH2, PolB, Fen1, and Ligase alone are sufficient to support RER.

## Discussion

### rNMPs in archaeal genomic DNA

Due to the high intracellular concentration of rNTPs relative to dNTPs, DNA polymerases incorporate rNTPs into genomic DNA at a much higher frequency than was previously appreciated. Importantly, rNMPs are now considered to be the most abundant non-canonical nucleotides in genomic DNA (6, 10, 12). Although rNMPs do not affect Watson-Crick base pairing, they do render DNA sensitive to strand cleavage and their presence affects the accuracy and efficiency of replication by altering the overall structure of DNA (13–17). Here we show that archaeal DNA polymerases incorporate ribonucleotides at a frequency of one per ∼1,500–2,000 nucleotides *in vitro* (Fig. 1*B*) and *in vivo* (Fig. 1*A*). Similar rNMP densities have been reported in budding and fission yeast and in mouse cells deficient in RER (10, 18, 22, 31). This rNMP burden in archaeal genomic DNA is quite substantial especially considering that the 2 Mb *Thermococcus* genome is ∼92% coding sequence (32). Using genetic and biochemical techniques, we identified and characterized the major pathway in *Thermococcus* that efficiently repairs embedded ribonucleotides.

The discovery that DNA polymerases frequently misincorporate rNTPs also led a number of groups to question whether ribonucleotides serve some potential cellular function, such as marking the nascent DNA strand for processes like mismatch repair (33). Functional or not, genomic rNMPs must be removed to protect genome stability so it is unexpected that the genes encoding essential RER components, including RNaseH2 and Fen1, can be deleted in archaea and yeast. Interestingly, RNaseH2 is essential in higher eukaryotes including mice and humans leading to embryonic lethality when deleted or severe disease when mutated (18, 19, 34). This begs the question: how do single-celled organisms lacking key RER components survive with so many incorporated ribonucleotides in their genomic DNA? The difference may lie in more complex DNA damage-response pathways that arrest growth and trigger cell death in higher eukaryotes. Additionally, *T. kodakarensis* is known to contain between 7 and 19 copies of its genome depending on the growth phase, which may be another genome protection mechanism (35). Finally, given that archaea naturally inhabit extreme environments, it is possible that outside of laboratory conditions, the lack of rNMP repair might result in detectable growth or other physiological defects. It is of interest to understand what additional mechanisms may exist in these organisms to tolerate unstable embedded ribonucleotides.

### RNaseH2 kinetics

RNaseH2 is the major enzyme required to initiate the archaeal RER pathway and *T. kodakarensis* lacks other redundant enzymes to initiate RER. RNaseH2 nicks at an rNMP in DNA rapidly (up to 450 ± 37 s^−1^; Table 2) regardless of the identity of the nucleobase. The rapid cleavage activity likely enables the repair of the as many as 1,000 rNMPs incorporated per replication cycle. However, the RNaseH2 steady-state turnover rate is slow compared with chemistry (0.06–0.17 s^−1^; Table 2) suggesting that other factors may be required for promoting RNaseH2 recycling to a new rNMP substrate. These properties have been described in other repair enzymes, such as human AP endonuclease I with a chemistry rate that is 425 times faster than its turnover rate (36). Slow turnover may ensure that nicked sites are not generated at a rate faster than the subsequent repair enzymes can process. RNaseH2 may remain bound at the nicked site to protect the nick or to recruit the rest of the RER machinery to complete processing.

The role for RNaseH2 in RER is highly conserved from archaea to eukaryotes despite the structural differences in these enzymes. Eukaryotic RNaseH2 is comprised of three essential subunits: RNaseH2A, RNaseH2B, and RNaseH2C. RNaseH2A is the catalytic subunit and has 26 and 36% identity to the monomeric RNaseH2 enzymes of *E. coli* and *T. kodakarensis*, respectively. The accessory subunits of the eukaryotic enzyme, RNaseH2B and RNaseH2C, are unique to eukaryotes. These additional subunits may serve structural roles or potentially modulate interactions with repair, replication, or other regulatory machinery (37). Additional subunits as well as PCNA may also confer processivity by stabilizing binding to DNA, which may be especially important for efficient repair in the context of their larger genomes (37, 38).

### The roles of PolB and PolD in RER

*Thermococcus* encodes two known DNA polymerases, PolB (the Family B polymerase) and PolD (the archaea-specific Family D polymerase), and the specific roles for each of these polymerases in DNA replication and repair are not fully understood. PolD subunits are essential genes (*tk_rs09520* and *tk_rs09525*), and the PolD enzyme is known to interact with other components of the replication machinery, while PolB is nonessential in several archaeal species and is not known to form complexes with other replisome proteins (39–42). Therefore, it has been proposed that PolD is the major leading and lagging strand replicative polymerase in archaea despite many of the replicative polymerases in other organisms belonging to family B. Our study sheds light on the division of labor between these two polymerases. The requirement for PolB rather than PolD in RER assays indicates the need for a strand displacing polymerase. In *Thermococcus*, only PolB possesses strong strand-displacement activity, a feature required for its role in both RER and Okazaki fragment maturation (43). However, because PolB is non-essential in most archaea expressing PolD, it is possible that weak strand displacement activity of PolD, or polymerases that have not yet been identified, carry out a low but sufficient level of repair in the absence of PolB (Fig. 5*C*). Additionally, the lack of known replisome-binding partners for PolB suggests that RER occurs outside of the assembled replisome, although the timing and coordination of repair remains to be determined.

Other DNA repair pathways such as base excision repair and nucleotide excision repair are also poorly understood in archaea. Given the extreme environments that these organisms inhabit, archaea likely accumulate substantial amounts of DNA damage that needs to be repaired to protect genome integrity. We speculate that the overall mechanism proposed here may be generalizable to other repair pathways as well, but making use of different enzymes to initiate each specific process. Future studies are needed to identify the initiating enzymes and determine whether PolB, Fen1, and Ligase are the primary repair enzymes required for these other types of repair as well.

### RER is evolutionarily conserved

**Figure 6.**
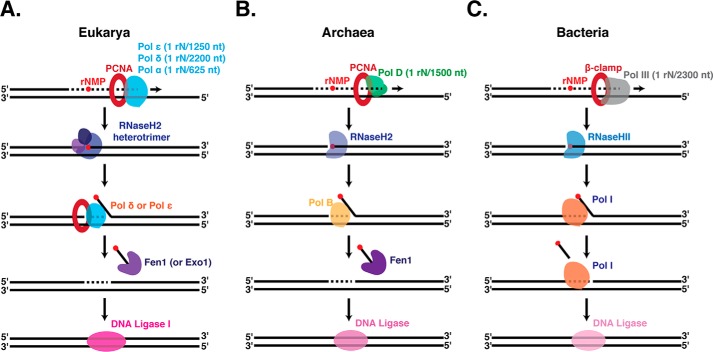
**Simplified models of ribonucleotide excision repair in Eukarya (*A*), Archaea (*B*), and Bacteria (*C*).** *A,* rNMPs are incorporated into eukaryotic genomic DNA by any of the replicative polymerases (Polϵ, Polδ, or Polα) with the incorporation frequencies shown (6, 9, 12). The RER pathway begins with incision by the heterotrimeric RNaseH2 followed by strand displacement synthesis by either of the replicative polymerases Polϵ or Polδ, flap cleavage by Fen1 or Exo1, and sealing by DNA ligase I. *B,* archaeal genomic DNA acquires rNMPs primarily through incorporation by the leading and lagging strand polymerase PolD at a rate of 1 rN in 1,500 nucleotides synthesized. Monomeric RNaseH2 nicks DNA at rNMP sites. Following cleavage, strand displacement synthesis by PolB creates a flap that is cleaved by Fen1 and DNA ligase seals the resulting nick. *C,* in bacteria, the replicative polymerase PolIII incorporates rNMPs in genomic DNA and monomeric RNaseHII nicks at these sites. PolI then fulfills two functions in RER by performing both strand displacement synthesis and flap cleavage. DNA ligase then seals the nick.

Our study revealed conservation of the overall mechanism of RER across archaea, bacteria, and eukaryotes (Fig. 6). In all domains of life, RER starts with nicking by RNaseH2 at the rNMP, strand displacement synthesis by a DNA polymerase, flap removal, and ligation. In archaea, single enzymes carry out each step of RER: rNMP nicking (RNaseH2), strand-displacement synthesis (PolB), flap cleavage (Fen1), and nick sealing (DNA ligase) (Fig. 6*B*). Furthermore, *Thermococcus* lack a redundant pathway to repair rNMPs. This contrasts with eukaryotes where redundant enzymes exist for several steps. Yeast RNaseH2 is the major enzyme to initiate RER yet the topoisomerase I-initiated RER pathway (21) acts as a redundant safeguard for genomic stability. Furthermore, in yeast RER either Polδ or Polϵ can carry out the strand displacement synthesis, and Fen1 or Exo1 can cleave the resulting flap (Fig. 6*A*) (11). Bacteria, on the other hand, combine several activities in a single enzyme where PolI functions as both the polymerase and the flap nuclease (Fig. 6*C*). Overall, the three domains of life have utilized a similar mechanism for excision of erroneous nucleotides, such as rNMPs, while using slightly different machinery to achieve it. Although we depict the RER pathway as a stepwise process of individual enzymes (Fig. 6), future studies will address how these different machineries are assembled, coordinated, and regulated.

## Experimental procedures

### Enzymes, oligonucleotides, and reagents

*Thermococcus* sp. 9°N PolD was purified as previously described (44). PCNA and RFC were prepared as previously described (43). All other proteins, nucleotides, buffers, and single-stranded M13mp18 DNA (ssM13) were from New England Biolabs, Inc. (Ipswich, MA). Oligonucleotides used in this study were labeled with either FAM or MAX fluorophores for detection and all were obtained from Integrated DNA Technologies (Coralville, IA) (Table 1).

### Cloning, expression, and purification of RNaseH2

The *Thermococcus* sp. 9°N RNaseH2 gene was codon optimized for expression in *E. coli*, constructed synthetically (Genscript, Piscataway, NJ), and cloned into pAII17 plasmid vector (45) cleaved with NdeI and BamHI to produce plasmid pESY. NEB T7 Express *E. coli* cells containing pESY were grown at 37 °C in 1 liter of LB media supplemented with 0.1 mg/ml of ampicillin. When the culture reached an *A*_600_ of 0.5, expression of 9°N RNaseH2 was induced by addition of 0.4 mm (final concentration) of isopropyl β-d-thiogalactopyranoside. Cells were allowed to grow for 3 h in the presence of isopropyl β-d-thiogalactopyranoside, and were collected by centrifugation at 4000 rpm for 15 min. Cell pellets were suspended in buffer A (20 mm Tris-HCl, pH 7.5, 0.1% Triton X-100, 5% glycerol) containing 0.25 m NaCl, and lysed by sonication and heating at 70 °C for 30 min. Centrifugation was used to remove cell debris. The supernatant was passed through a DEAE column and flow through was collected and diluted with buffer A to 50 mm NaCl. Diluted flow through was loaded onto a Heparin TSK column, pre-equilibrated with buffer A containing 50 mm NaCl, and eluted with a buffer A gradient from 50 mm to 1 m NaCl. Peak fractions were identified by SDS-PAGE analysis, pooled, and dialyzed to 10 mm Tris-HCl, pH 7.5, 100 mm KCl, 0.1 mm EDTA, 0.1% Triton X-100, and 50% glycerol.

### Strains

*T. kodakarensis* (Tko) strains were grown as previously described (40). Tko strains deleted for RNaseH2 (ΔRNaseH2) and Fen1 (ΔFen1) were constructed as described,^3^ and strains deleted for PolB were generated previously (40).

### Alkaline hydrolysis and alkaline-agarose electrophoresis of RNA-DNA hybrids

Ribonucleotides in DNA are visualized using alkaline-agarose electrophoresis. Treatment with NaOH fragments DNA 3′ to an embedded rNMP, resulting in DNA that migrates faster by alkaline-agarose electrophoresis. 2 m NaOH (or 5 m NaCl as negative control) was added to DNA at 0.3 m final concentration and heated at 55 °C for 2 h to hydrolyze at ribonucleotide positions in DNA. 6× Purple loading dye (New England Biolabs, Inc.) was added to samples before loading onto a 1% alkaline-agarose gel (1% agarose, 1 mm EDTA, 50 mm NaOH). The gel was run at 5 V for 16 h, stained with SYBR Gold (Thermo Fisher Scientific), and visualized on a UV imager.

### Analysis of rNMP incorporation by 9°N PolB and PolD

The frequency of PolB and PolD rNMP incorporation was determined by primer extension on a single-stranded M13 substrate followed by alkali treatment and analysis by alkaline-agarose gel electrophoresis as described (46). Primer extension reactions (50 μl) were assembled by mixing primed ssM13 DNA substrate (30 nm) (45), ThermoPol Buffer (1 ×) (20 mm Tris-HCl, 10 mm (NH_4_)_2_ SO_4_, 10 mm KCl, 2 mm MgSO_4_, 0.1% Triton X-100, pH 8.8, at 25 °C), additional MgSO_4_ (10 mm), [α-^32^P]dCTP (as a radioactive tracer), 9°N PCNA (40 nm as a trimer), and 9°N RFC (80 nm as a dimer). Reactions were performed with either dNTPs (100 μm) or a physiological concentration of rNTPs and dNTPs of 16 μm dATP, 14 μm dCTP, 12 μm dGTP, 30 μm dTTP, 3000 μm ATP, 500 μm CTP, 700 μm GTP, and 1700 μm UTP (11). Reactions were initiated with PolB or PolD (200 nm) and incubated at 65 °C for 30 min. Reactions were resolved by neutral or alkaline-agarose gel electrophoresis, exposed to a storage phosphor screen (GE Healthcare) and imaged on a Typhoon 9400 scanner. ImageQuant software was used to plot fragment distribution.

### Steady-state RNaseH2 cleavage kinetics

The rGMP RER substrate used for steady-state kinetics was prepared by annealing the 50-nt 5′-FAM/3′-MAX rGMP oligonucleotide (1 μm) (Table 1) to the 50-nt unlabeled complement (1.5 μm) (Table 1) in ThermoPol II buffer (20 mm Tris-HCl, 10 mm (NH_4_)_2_ SO_4_, 10 mm KCl, 0.1% Triton X-100, pH 8.8, at 25 °C) by heating to 95 °C for 5 min and cooling to room temperature. A 600-μl dilution of the rG DNA substrate (50 nm) was made in ThermoPol buffer (1 × concentration) and a 600-μl dilution of the *Thermococcus* sp. 9°N RNaseH2 (10 nm) in ThermoPol buffer (1 ×). The two solutions were mixed in a RQF instrument (KinTek Corp., Snow Shoe, PA) set to 62.5 °C with a circulating water bath to achieve a final 60 °C reaction temperature. Time points were mixed for 0.004–10 s and quenched with 50 mm EDTA. After mixing, the final reaction concentrations were 5 nm RNaseH2 and 25 nm DNA substrate in 1× ThermoPol buffer for a 5-fold excess of DNA substrate. The no enzyme (0 s) control was performed by rapidly mixing 1× ThermoPol buffer with the DNA substrate in the RQF for 10 s. To ensure that steady-state conditions had been achieved in these experiments, control experiments were performed with a final reaction condition of 5 nm RNaseH2 and 50 nm DNA substrate in 1× ThermoPol buffer (10-fold excess of substrate to enzyme) (data not shown). Reaction products were separated by capillary electrophoresis using a 3730xl Genetic Analyzer (Applied Biosystems) and the fluorescent peaks were analyzed using Peak Scanner software version 1.0 (Applied Biosystems). The concentration of 21-nt FAM product was graphed as a function of time and fit to Equation 1 using the nonlinear regression software Kaleidagraph (Synergy Software). The steady-state rate (*k*_ss_) was obtained by dividing the linear rate (*k*_2_) by the amplitude (A). All kinetic assays were performed at least three times for reproducibility.

(Eq. 1) $$\left[\text{Product}\right]=A\left(1-\exp\left(-k_{\text{obs}}\times t\right)\right)+k_{2}\times t$$

### Pre-steady-state RNaseH2 cleavage kinetics

The rGMP, rAMP, rCMP, and rUMP substrates were prepared by annealing the 50-nt 5′-FAM rNTP oligonucleotide (1 μm) to its 50-nt unlabeled complement (1.5 μm) (Table 1) in ThermoPol II buffer by heating to 95 °C for 5 min and cooling to room temperature. A 300-μl aliquot of 90 nm RNaseH2 and 30 nm DNA substrate was mixed in 1× ThermoPol II buffer to prebind enzyme to DNA. This mixture was rapidly mixed with 1× ThermoPol II buffer containing 4 mm MgSO_4_ in the RQF instrument to trigger the reaction. Time points were mixed for 0.002–0.2 s and quenched with 50 mm EDTA. After mixing, the final reaction concentrations were 45 nm RNaseH2, 15 nm DNA substrate, 2 mm MgSO_4_ in 1× ThermoPol II buffer. The no enzyme (0 s) control was performed by rapidly mixing 1× ThermoPol II buffer with the DNA substrate-RNaseH2 mixture in the RQF for 5 s to show that the enzyme is fully chelated and has no activity in the absence of MgSO_4_. To ensure that pre-steady-state conditions had been achieved, control experiments were performed where the enzyme was in 6-fold excess to DNA substrate with a final reaction condition of 90 nm RNaseH2 and 15 nm DNA substrate (data not shown). Reaction products were separated by capillary electrophoresis and analyzed as described above. The concentration of 21-nt FAM product was graphed as a function of time and fit to Equation 2 using the nonlinear regression software Kaleidagraph (Synergy Software) to obtain the pre-steady-state cleavage rate. All kinetic assays were performed at least three times for reproducibility.

(Eq. 2) $$\left[\text{Product}\right]=A\left[1-\exp\left(-k_{\text{obs}}\times t\right)\right]$$

### Tko extract RER assays

The rG RER substrate was prepared by annealing the 44-nt 5′-MAX oligonucleotide (400 nm), 90-nt 3′-FAM rG oligonucleotide (410 nm), and ssM13mp18 (480 nm) in ThermoPol buffer II by heating to 95 °C for 5 min and cooling to room temperature. *T. kodakarensis* (Tko) KW128 (wild-type) and mutant strains (ΔRNaseH2, ΔFen1, and ΔPolB) were grown as previously described (40). Tko cell extracts were prepared by suspending cell paste (1 g) in 1 ml of Buffer A (20 mm Tris-HCl, pH 7.5, 50 mm NaCl). Suspended cells were sonicated for 2 min and centrifuged for 10 min at 14,000 × *g* to remove cell debris. The extract supernatant was mixed with glycerol to 50% final concentration, and stored at −20 °C. All extracts were normalized by DNA ligase activity as all strains contained the essential gene for DNA ligase. The rG RER substrate (15 nm) was incubated with Tko extracts as well as 1× ThermoPol buffer, 1 mm ATP, and 0.1 mm dNTPs at 60 °C over a time course from 0 to 60 min. Reactions were quenched with 50 mm EDTA and products were separated by capillary electrophoresis. The conversion of the 90-nt FAM oligonucleotide to the 134-nt MAX/FAM dual-labeled repair product was quantified for each Tko extract at the 60-min time point using Peak Scanner software as described above. Three biological replicates were performed for the extract experiments. For RNaseH2 assays (Fig. 1*C*), Tko extracts (wild-type and ΔRNaseH2) were prepared as described above and incubated with 10 nm rG 50-nt FAM/MAX DNA substrate, 1 mm ATP, 0.1 mm dNTPs, and 1× ThermoPol buffer at 60 °C over a time course from 0 to 20 min. Extracts were complemented with purified proteins (1 nm) (supplemental Fig. S4). Reactions were quenched with 50 mm EDTA, products were separated by capillary electrophoresis, and results were quantified using Peak Scanner software.

### RER in vitro reconstitution

Repair reactions were performed by mixing the rG RER substrate (10 nm) with purified *Thermococcus sp.* 9°N proteins at the following concentrations: 1 nm Fen1, 1 nm Ligase, 1 nm PolB, 1 nm PolD, 20 nm PCNA, 40 nm RFC. All reactions were performed in 1× ThermoPol buffer with 1 mm ATP and 0.1 mm dNTPs. The RER reactions were carried out at 60 °C over a time course from 0 to 30 min with different protein components omitted from the mix to assay their role on RER. The conversion of the 90-nt FAM oligonucleotide to the 134-nt MAX/FAM dual-labeled repair product was quantified for each reaction condition at the 30-min time point using Peak Scanner software as described above. Experiments were performed three times for reproducibility.

## Author contributions

M. R. H. designed and conducted experiments, analyzed results, and wrote the manuscript. B. W. B. constructed and grew *Thermococcus* strains. T. J. S. analyzed results and wrote the manuscript. A. F. G. designed experiments, analyzed results, and wrote the manuscript. All authors analyzed the results and approved the final version of the manuscript.

## Supplementary Material

Supplemental Data

## Footnotes

3 Burkhart, B. W., Cubonova, L., Heider, M. R., Kelman, Z., Reeve, J. N., and Santangelo, T. S. (2017) The GAN exonuclease, or the flap endonuclease Fen1 and RNase HII are necessary for viability of *Thermococcus kodakarensis. J. Bacteriol.* 10.1128/JB.00141-17.

## Footnotes

2 The abbreviations used are:

rNMP: ribonucleotide monophosphate
Tko: *Thermococcus kodakarensis*
9°N: *Thermococcus* sp. 9°N
PolB: Family B DNA polymerase
PolD: Family D DNA polymerase
Fen1: flap endonuclease 1
PCNA: proliferating cell nuclear antigen
RFC: replication factor C
FAM: 5-carboxyfluorescein
nt: nucleotide(s)
RQF: rapid chemical quench instrument
CE: capillary electrophoresis
RER: ribonucleotide excision repair.
