# Supplementary material for: Defining the RNaseH2 enzyme-initiated ribonucleotide excision repair pathway in Archaea
Source: J Biol Chem. 2017 Apr 3;292(21):8835–45. doi: 10.1074/jbc.M117.783472 (PMC5448109; doi:10.1074/jbc.M117.783472)
Supplement: Supplemental Data [file supp_292_21_8835__index.html]

Defining the RNaseH2 enzyme-initiated Ribonucleotide Excision Repair Pathway in Archaea — Defining the RNaseH2 enzyme-initiated ribonucleotide excision repair pathway in Archaea — Archaeal ribonucleotide excision repair — Supplemental Data 

# Defining the RNaseH2 enzyme-initiated ribonucleotide excision repair pathway in Archaea

## Supplemental Data

- Supplemental Data (.pdf, 3.8 MB) - Supplemental Data
