# Supplementary material for: Defining the RNaseH2 enzyme-initiated ribonucleotide excision repair pathway in Archaea
Source: J Biol Chem. 2017 Apr 3;292(21):8835–45. doi: 10.1074/jbc.M117.783472 (PMC5448109; doi:10.1074/jbc.M117.783472)
Supplement: Supplemental Data [file 10.1074_M117.783472_jbc.M117.783472-1.pdf]

Defining the RNaseH2 enzyme-initiated Ribonucleotide Excision Repair Pathway in Archaea

Margaret R. Heider[1], Brett W. Burkhart[2], Thomas J. Santangelo[2], and Andrew F. Gardner[1]

[1]From New England Biolabs, Inc., Ipswich, Massachusetts 01938, [2]From Department of Biochemistry and Molecular Biology, Colorado State University, Fort Collins, CO 80521

Supplemental Data

Figure S1. 9°N RNaseH2 steady-state kinetics of rG, rA, rU and rC cleavage.

Figure S2. 9°N RNaseH2 pre-steady-state kinetics of rG, rA, rU and rC cleavage.

Figure S3. 9°N RNaseH2 does not cleave 134 nt sealed product confirming removal of ribonucleotide.

Figure S4. Addition of purified protein restores repair in Tko deletion extracts.

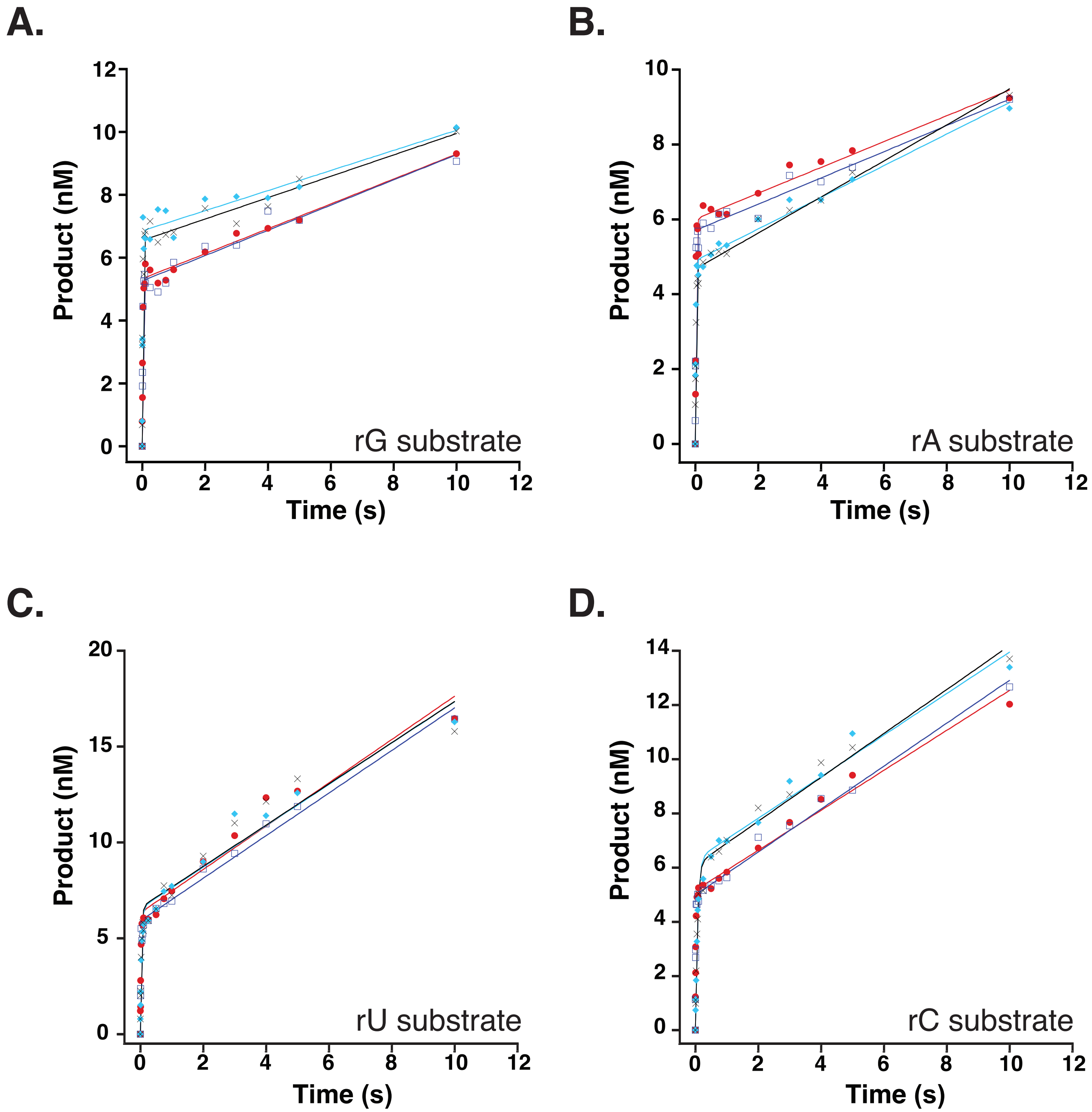

Figure S1. 9°N steady-state kinetics of rG, rA, rU, and rC cleavage. For steady-state kinetics, a 5-fold excess of RER substrate was rapidly mixed with purified *Thermococcus* sp. 9°N RNaseH2 in an RQF instrument at 60°C. The reaction was quenched with 50 mM EDTA. The conversion of the 50-nt, substrate to 5'FAM and 3'MAX cleavage products was monitored over time using CE. The yield of 5’ FAM product was graphed as a function of time and fit to obtain k_{ss} for each RER substrate. Four replicates are shown for each rNMP substrate: rG (A), rA (B), rU (C), rC (D).

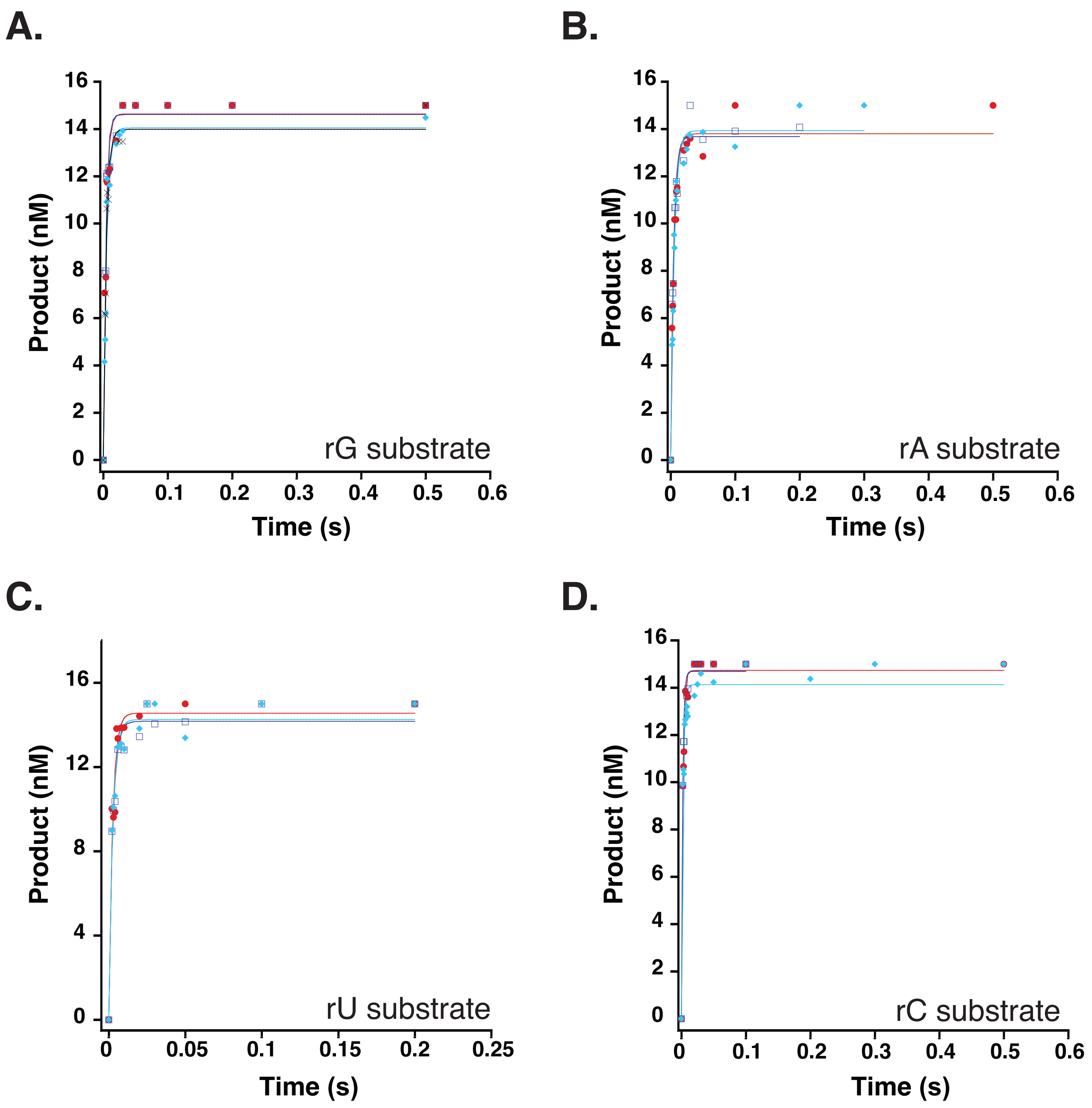

Figure S2. 9°N pre-steady-state kinetics of rG, rA, rU, and rC cleavage. For pre-steady-state kinetics, a 3-fold excess of purified *Thermococcus* sp. 9°N RNaseH2 was rapidly mixed with RER substrates in an RQF instrument at 60°C. The reaction was quenched with 50 mM EDTA. The conversion of the 50-nt, substrate to 5'FAM and 3'MAX cleavage products was monitored over time using CE. The yield of 5' FAM product was graphed as a function of time and fit to obtain $k_{cleavage}$ for each RER substrate. Four replicates are shown for each rNMP substrate: rG (A), rA (B), rU (C), rC (D).

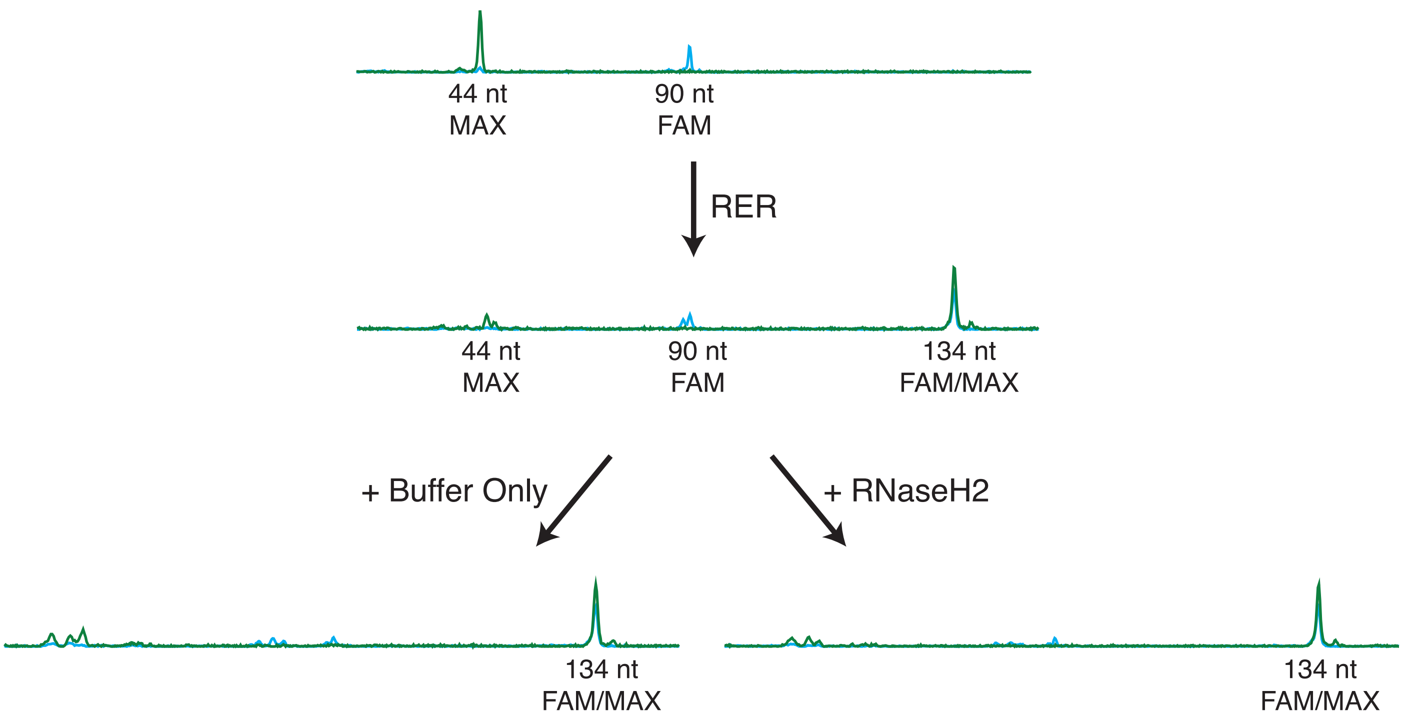

Figure S3. Repaired M13 substrate does not contain a ribonucleotide. 9°N RNaseH2 does not cleave 134 nt sealed product confirming removal of ribonucleotide.The substrate depicted in Figure 3 is incubated with purified *Thermococcus* sp. 9°N proteins including PCNA, RFC, PolB, PolD, Fen1, and DNA ligase for 30 minutes at 60°C for complete conversion to 134 nt sealed product. After 30 minutes, 9°N RNaseH2 or an equal volume of 1X ThermoPol buffer was added and incubated over a time course from 0-60 minutes at 60°C. The 60 minute time point is shown. No signal for 44 nt MAX or 90 nt FAM oligonucleotide cleavage products was observed at any time point.

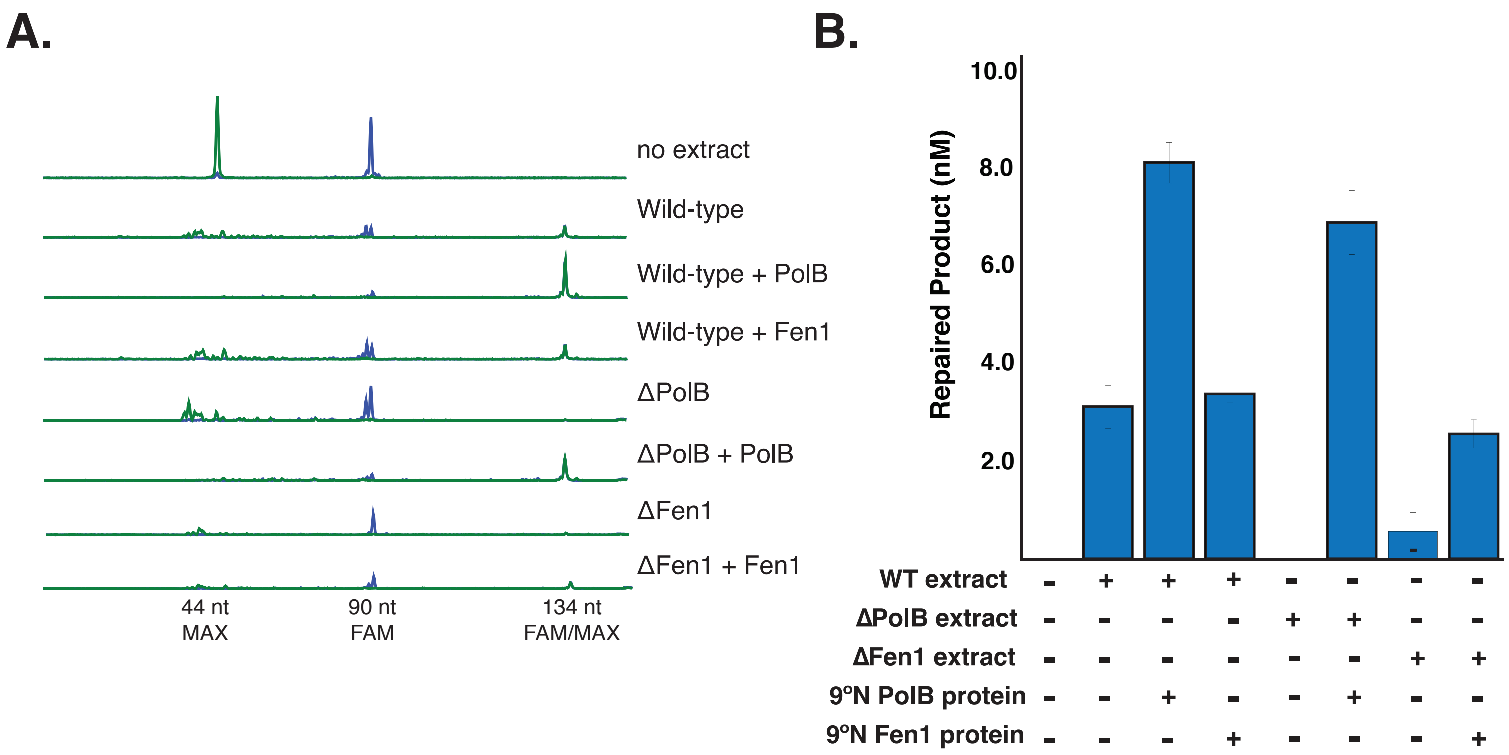

Figure S4. Addition of purified protein restores repair in Tko deletion extracts. (a) Representative CE traces for RER assay with Tko extracts. The substrate depicted in Figure 3 was incubated with Tko extracts for 0 to 60 minutes at 60°C and 60 minute time points are shown. Purified 9°N proteins were added to extracts at the start of reactions at 1 nM final concentration. (b) The amount of repaired product at 60 minutes was quantified for each extract. The data shown are the average of four independent experiments with standard deviation.
